# A novel framework for rapid diagnosis of COVID-19 on computed tomography scans

**DOI:** 10.1007/s10044-020-00950-0

**Published:** 2021-01-22

**Authors:** Tallha Akram, Muhammad Attique, Salma Gul, Aamir Shahzad, Muhammad Altaf, S. Syed Rameez Naqvi, Robertas Damaševičius, Rytis Maskeliūnas

**Affiliations:** 1 Department of EE, COMSATS University Islamabad, Wah Campus, Pakistan; 2 Department of Computer Science, HITEC University Taxila, Rawalpindi, Pakistan; 3 Department of Radiology, Shifa International Hospital, Islamabad, Pakistan; 4 Department of EE, COMSATS University Islamabad, Abbottabad Campus, Pakistan; 5 Faculty of Applied Mathematics, Silesian University of Technology, Gliwice, Poland; 6 Department of Applied Informatics, Vytautas Magnus University, Kaunas, Lithuania

**Keywords:** Covid19, Features extraction, Features selection, Features classification

## Abstract

Since the emergence of COVID-19, thousands of people undergo chest X-ray and computed tomography scan for its screening on everyday basis. This has increased the workload on radiologists, and a number of cases are in backlog. This is not only the case for COVID-19, but for the other abnormalities needing radiological diagnosis as well. In this work, we present an automated technique for rapid diagnosis of COVID-19 on computed tomography images. The proposed technique consists of four primary steps: (1) data collection and normalization, (2) extraction of the relevant features, (3) selection of the most optimal features and (4) feature classification. In the data collection step, we collect data for several patients from a public domain website, and perform preprocessing, which includes image resizing. In the successive step, we apply discrete wavelet transform and extended segmentation-based fractal texture analysis methods for extracting the relevant features. This is followed by application of an entropy controlled genetic algorithm for selection of the best features from each feature type, which are combined using a serial approach. In the final phase, the best features are subjected to various classifiers for the diagnosis. The proposed framework, when augmented with the Naive Bayes classifier, yields the best accuracy of 92.6%. The simulation results are supported by a detailed statistical analysis as a proof of concept.

## Introduction

**Fig. 1:**
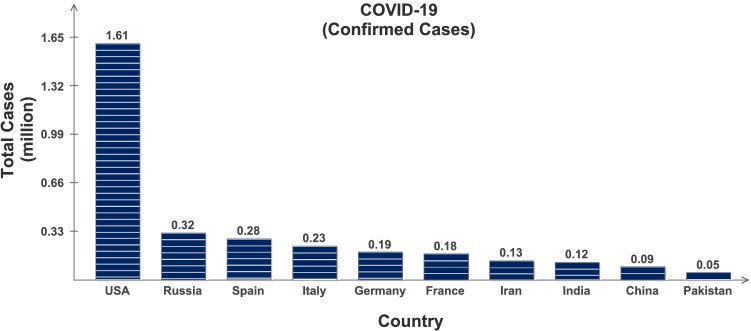
Confirmed COVID-19 cases in 10 selected countries: Retrieved on May 19, 2020 from www.worldometer.info

**Fig. 2:**
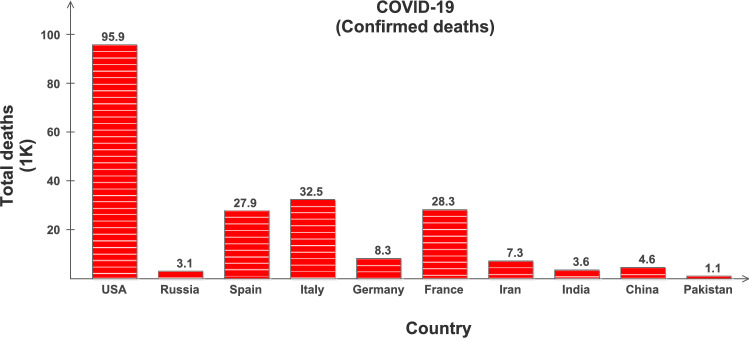
Confirmed deaths in 10 selected countries: Retrieved on May 19, 2020 from www.worldometer.info

Coronavirus Disease 2019 (COVID-19) is highly contagious and has rapidly spread globally infecting almost all the countries with millions of positive cases and more than 0.4 million deaths [1], and is continuously on the rise; see Table 1 and Figs. 1 and 2. The key factor to limit this pandemic situation is the early testing and diagnosis. However, due to its pandemic nature, quick collection and testing of samples from the suspected patients is a challenging issue for clinical management. Its early detection is possible with Nucleic Acid Amplification Tests (NAAT), such as reverse transcription polymerase chain reaction (RT-PCR) [2], which is required to be interpreted by trained clinical laboratory personnel [3]. The initial symptoms of COVID-19 are fever, fatigue and dry cough, and it predominantly affects lungs. The affected lobes with ground glass changes and/or consolidations etc. can be recorded in chest radiology images [4, 5]. This is why the clinicians worldwide are using chest X-rays (CXR) and computed tomography (CT) images as an alternative and fast method for the screening and diagnosis of the COVID-19, especially supported by the fact that the RT-PCR method may take several hours to complete the process [6].

Now due to the rise of this pandemic situation, thousands of people daily undergo CXR and CT scan for screening of COVID-19. This has overburdened the radiologist leading to their decreased productivity [7] in the proper detection of suspicious abnormalities [8]. In the case of contagious diseases, this backlog of radiological studies cannot be afforded; however, in the case of chronic and slow diseases such studies may be delayed. For detailed analysis of the errors and discrepancies in radiology diagnosis, the interested readers are referred to [9–17]. Artificial intelligence (AI) and computer vision system play an important role in classifying different complex structures found in the medical images [18–21] and can be used in the computer aided diagnosis tools. Therefore, AI is being researched for radiological diagnosis since long and has proven quite successful in the cases of breast screening [22], diagnosis and quantification of emphysema severity [23], tuberculosis detection [24] and claims of detection of diagnosis idiopathic pulmonary fibrosis with similar accuracy to a human reader [25].

During the past year, several imaging-based diagnosis techniques of COVID-19 backed by AI and machine learning have been presented, along with their correlation with the RT-PCR [26, 27]. CT and CXR images are processed for the detection of pneumonia like imaging features using AI techniques. In [28], CXR images and deep convolutional neural networks (CNNs) are used to diagnose COVID-19. The models used are ResNet 50, Inception V3 and a hybrid approach based on Inception-ResNetV2, claiming accuracies of $98\%, 97\%$ and $87\%$, respectively. In another approach [29], COVID-19 and bacterial and viral pneumonia are diagnosed and classified into negative or positive by using X-ray radiographs. The approach makes use of GoogleNet as a deep transfer model, claiming $100\%$ accuracy. In another approach [30], the authors have claimed $86.7\%$ accuracy in diagnosing the early signs of COVID-19, using chest CT images with deep learning . Authors in [31] propose to use two-dimensional (2D) and three-dimensional (3D) deep learning models combination and claim an accuracy of $98.2\%$ and specificity of $92.2\%$ with chest CT images. In [32], the authors combined the Inception CNN with Marine Predators algorithm to select the most relevant features from COVID-19 X-ray images, achieving an accuracy of 98.7%. In another study, the deep learning methods are used to extract COVID-19 graphical features and provide clinical diagnosis quite ahead of the pathogenic test helping in timely control of the spread. The authors claim a rather constrained accuracy of $85.2\%$ [10].

On the one hand, it is widely accepted that the diagnoses based on CXR are not as efficient as those based on the CT scans [11]; on the other hand, however, the accuracies reported in the literature for the former surprisingly exceed those for the CT scans. Furthermore, these systems normally give binary decision of either negative or positive, and do not incorporate any qualitative analyses based on the recommendations of Radiological Society of North America (RSNA) [33, 34]. Hence, it may be concluded that most of the available methods are either not sufficiently reliable, or achieve a constrained diagnosis efficiency. Especially for contagious diseases, such as COVID-19, there is still space for a thorough framework that could address the aforementioned discrepancies.

**Table 1:** Covid-19 statistics: Retrieved on May 19, 2020 from www.worldometer.info

Country	Total cases	Total deaths	Total recovered	Active cases	Critical cases
USA	1,612,450	95,923	374,177	1,142,350	17,964
Russia	317,554	3099	92,681	221,774	2300
Spain	280,177	27,940	196,958	55,219	1152
Italy	228,006	32,486	134,560	60,960	640
France	181,826	28,215	63,858	89,753	1745
Germany	178,918	8282	158,000	12,636	1016
China	82,967	4634	78,249	84	8
Iran	129,341	7249	100,564	21,528	2655
India	118,226	3584	48,553	66,089	
Pakistan	48,091	1017	14,155	32,919	111

In this research work, the CT scan images and CXR images are processed for the detection of radiological signs of COVID-19 using computer vision and AI techniques and classified these images as per the RSNA recommendations using a novel machine learning approach. The proposed technique is capable of minimizing inter-observer variability in image interpretation among the radiologists and hence subjectivity due to difference in experience by qualitative analysis. The system is also capable of picking up very subtle or early findings that can be missed by a radiologist. The solution is a combination of preprocessing stages, especially designed to extract the information using a set of selected feature extraction techniques. The main contributions of the proposed framework are summarized as follows:

1. A novel entropy-based fitness optimizer function is implemented, which selects the chromosomes with maximum information. The only chromosome with maximum fitness value is selected to get the sub-optimal solution in the minimum number of iterations.
2. To conserve maximum information and to obliterate the redundant features at the initial level, a preliminary selection process is initiated on each feature set using the entropy-controlled fitness optimizer.
3. To exploit the complementary strength of all features, a feature fusion approach is utilized which combines all the competing features to generate a resultant feature vector.

The rest of the manuscript is organized as follows: Sect. 2 presents the two commonly used imaging analysis for COVID-19. Datasets and their collection are given in Sect. 3, followed by a detailed description of the proposed framework in Sect. 4. The results and statistical analysis are presented in Sects. 5 and 6, respectively. We conclude the manuscript in Sect. 7.

## CT and CXR imaging analysis for COVID-19

**Fig. 3:**
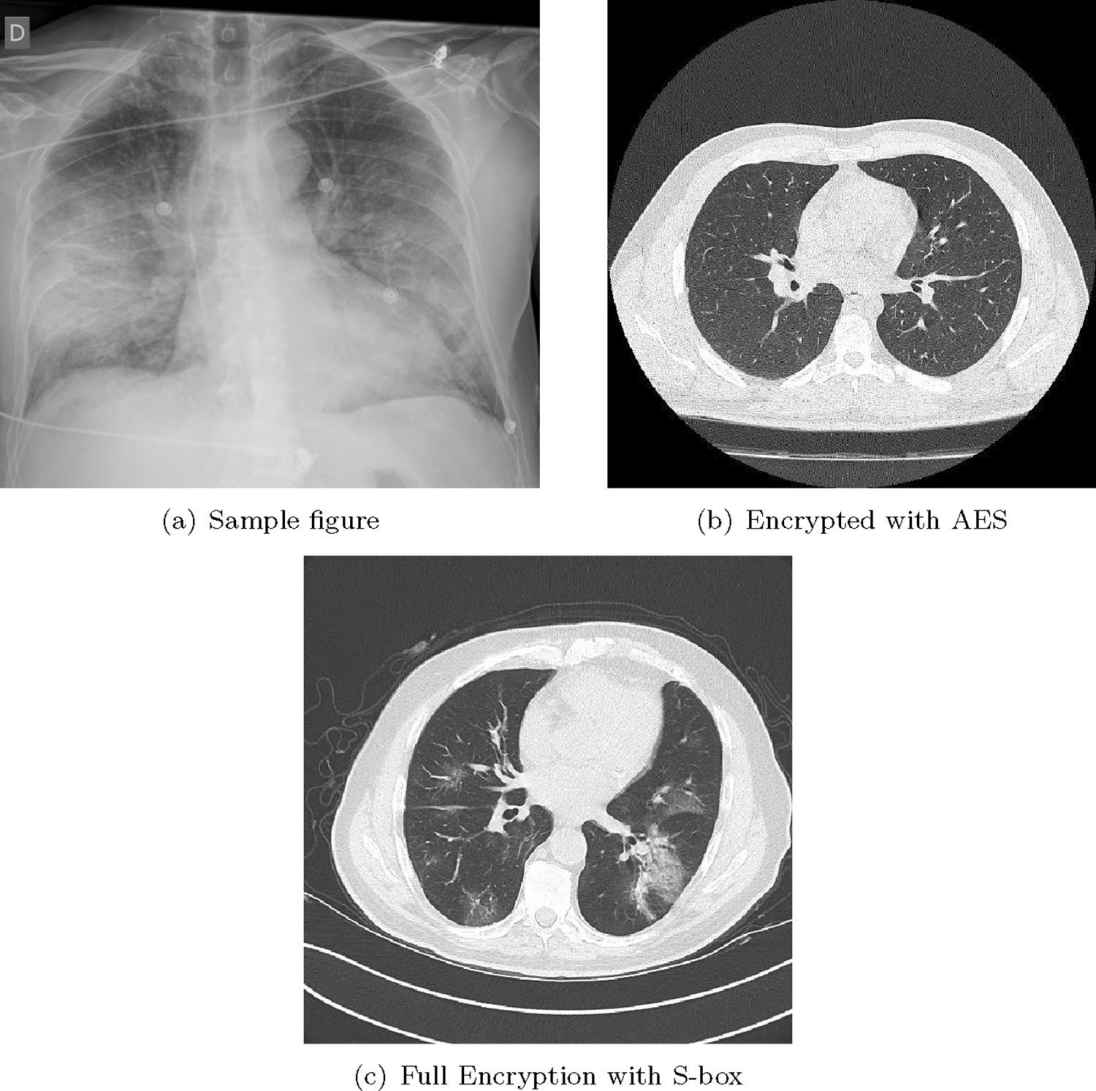
CXR and CT images of COVID-19 patients

COVID-19 can be detected with Nucleic Acid Amplification Tests, such as RT-PCR at very early stage [3] which is the gold standard as yet. Some studies have shown that imaging should be discouraged as primary screening tool, because these may suffer from selection bias (from inter observer variability among radiologists) with the claims that it is ten times less sensitive and less specific as compared to RT-PCR [35]. This implies that it can be negative in the early stages of the disease, and imaging features can overlap with many other infectious and noninfectious disease processes. However, in China, the chest CT has proven to have relatively higher sensitivity for COVID-19 as compared to the initial RT-PCR from swab samples [27], possibly due to the high sensitivity of CT images to lung lesion even before RT-PCR [36, 37]. As the RT-PCR takes more time, that is at least 6 h, imaging was a much faster and readily available screening tool in the surge of patients during the pandemic situation especially where RT-PCR were not available; therefore, it has played an important role in the risk stratification and screening for COVID -19. Chest radiographs are less sensitive as compared to chest CT and can give negative diagnose in case of early or mild infection, but can be used as a first line imaging modality [38]. On CXR, the findings may be airspace opacities or Ground Glass Opacities (GGO) mostly distributed in bilateral, peripheral and lower zone [38, 39]. In the early stages of disease, the CT images may be either negative or show GGO only, while at progressive stage increased GGO and crazy paving can appear [35]. The representative CXR and CT scan images of COVID-19 are shown in Fig. 3. In Fig. 3a, CXR shows Bilateral Ground Glass alveolar consolidation with peripheral distribution, which is very clear and can be seen easily, but this may not always be the case, specifically in the early stages of infection. In Fig. 3b, c, two CT images are given, showing air space consolidation and GGO. The changes are much clearly visible in Fig. 3c, while in Fig. 3b the GGO can be misinterpreted with motion blur. This is important to note as it can affect the efficient implementation of an intelligent diagnosis system. It is important to note that many respiratory viruses, such as influenza, organizing pneumonia and connective tissue disorders, can cause pneumonia like changes on both chest radiograph and CT similar to that of COVID-19, and therefore, their proper interpretation and differentiation from COVID-19 is a challenging issue [40, 41]. In order to address such ambiguities, RSNA has recommended statements on reporting CXR and CT finding related to COVID-19 [33, 34]. It is important to follow these recommendations to avoid any misinterpretation.

## Dataset collection

We collected pneumonia chest CT scans of 35 subjects diagnosed positive of COVID-19 from RadioPaedia image database [2]. Links to some public domain websites are also given for verification as follows:

1. Case 1—https://radiopaedia.org/cases/covid-19-pneumonia-2
2. Case 3—https://radiopaedia.org/cases/covid-19-pneumonia-3
3. Case 4—https://radiopaedia.org/cases/covid-19-pneumonia-4
4. Case 5—https://radiopaedia.org/cases/covid-19-pneumonia-7
5. Case 8—https://radiopaedia.org/cases/covid-19-pneumonia-8
6. Case 15—https://radiopaedia.org/cases/covid-19-pneumonia-23
7. Case 18—https://radiopaedia.org/cases/covid-19-pneumonia-10
8. Case 20—https://radiopaedia.org/cases/covid-19-pneumonia-27

All the patients have their RT-PCR COVID-19 test positive and have COVID19-pneumonia. The patients’ history is also provided in the given website along with their detailed travel history. For the healthy scans, we approached [29], so that the model utilized is efficiently trained, Fig. 3a. This figure portrays healthy/normal scans in the top row, while the COVID-19 pneumonia scans are provided in the bottom row. To train the classifier, features extracted from a total of 3500 COVID19-pneumonia and 2400 normal chest CT scans are utilized from the provided link, and are resized into $512\times 512$ resolution.

## Proposed methodology

**Fig. 4:**
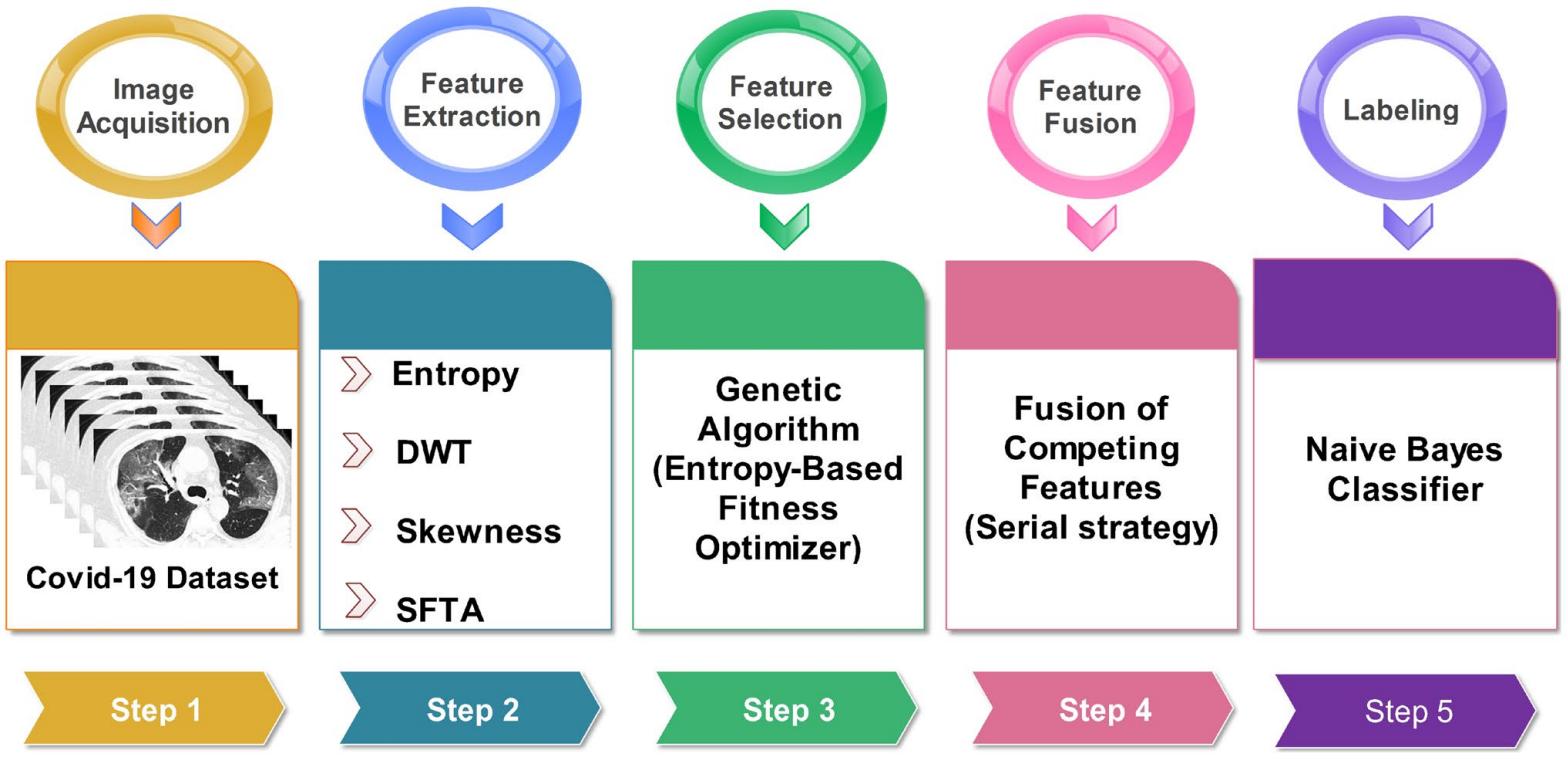
Proposed framework of COVID19 prediction using CT scans

Early methods of machine learning utilize either sole or hybrid approaches for feature extraction. Though both methods have their advantages and drawbacks, generally fused feature space has more capacity to retain the dexterous features. Due to this flexibility, the hybrid approaches have gained much popularity among the researchers working in the area of computer vision. However, selection of the most appropriate feature extraction technique is quite a sensitive task, which needs to be handled carefully, otherwise, it may result in feature redundancy and, therefore, increased correlation. In this work, we utilized four different techniques—belongs to two different categories, statistical and texture. Two feature families are not considered, color and shape, because of their limited impact and significance in this application. The proposed framework, Fig. 4, is the subject of discussion in the following subsections.

### Discrete wavelet transform features

The rationale behind selecting the discrete wavelet transform (DWT) for texture feature extraction is its ability to be invariant to translation, scaling and rotation. Further, in DWT, the contours can be requited from the coarsest to the finer scale, enabling the formulation to handle noise effectively. The 2D wavelet decomposition for images is similar to the 1D decomposition, in which the 2D wavelets basis $\Psi _{l,m(t)}$ and scaling basis $\phi _{m}(t)$ are obtained by taking the tensor products of 1D wavelets and scaling functions. For a 2D image, the DWT performs critical subsampling along both rows and columns, and these subbands information is utilized in the next level decomposition. The followed approach utilizes filter banks, described as:

1 $$\begin{aligned} \alpha _l(k)&=   \sum _{u}6h_0(u-2k)\alpha _{l+1}(u) \end{aligned}$$

2 $$\begin{aligned} \varsigma _l(k)&=   \sum _{u}h_1(u-2k)\alpha _{l+1}(u) \end{aligned}$$

The lowpass or averaged coefficients $\alpha _l(k)$ are created by half-band lowpass filter $h_o$, whereas the highpass or detailed coefficients $\varsigma _l(k)$ are created by half-band highpass filter $h_1$. From the equations, it can be observed that filtering $\alpha _{l+1}$ with $h_0$ and $h_1$ produces $\alpha _l(k)$ and $\varsigma _l(k)$, followed by decimation by a factor of 2.

To compute DWT coefficients for two levels, two-stage filter banks are required. The initial scale, $l+1$, in terms of $\alpha _{l+1}$ is the original signal, which after one level of decomposition produces highpass $\varsigma _l$ and lowpass coefficients $\alpha _l$. A batch of COVID-19 chest CT scans are represented by $\{x_1, x_2, x_3,\dots , x_n\} \subseteq {X(m,n)} \in {\mathbb {R}}^{\{m,n\}}$, where *m* and *n* are the rows and columns, respectively. Initially, both filters $H_0(\omega )$ and $H_1(\omega )$ are applied on $x_1{m,n}$ to generate a pair of images with both low and high frequencies. Afterward, the filtered images are sub-sampled by a factor of 2 and are forwarded to the next series of filters along the columns. The decimation by a factor of 2 is again carried out after filtration process in the columns. A single column decomposition generates four subband images, $\{LL, LH, HL, HH\}$ of size $\frac{M}{2}, \frac{N}{2}$. The whole computation is performed to generate set of features:

3 $$\begin{aligned} LL^l(u,v)&=   \sum _{m}\sum _{n}~h_0(m-2u)~h_0(n-2v)~.~LL^{l+1}(m,n), \end{aligned}$$

4 $$\begin{aligned} HL^l(u,v)&=   \sum _{m}\sum _{n}~h_0(m-2u)~h_1(n-2v)~.~LL^{l+1}(m,n), \end{aligned}$$

5 $$\begin{aligned} LH^l(u,v)&=   \sum _{m}\sum _{n}~h_1(m-2u)~h_0(n-2v)~.~LL^{l+1}(m,n), \end{aligned}$$

6 $$\begin{aligned} HH^l(u,v)&=   \sum _{m}\sum _{n}~h_1(m-2u)~h_1(n-2v)~.~LL^{l+1}(m,n), \end{aligned}$$

The lowpass *L* and highpass *H* filters are represented by the alphabet letters on the sub images.

### Extended segmentation-based fractal texture analysis (ESFTA)

As discussed earlier, texture features play much more significance role in the recognition process compared to other set up of features including shape and color. Therefore, in this work, we are employing our existing work [42] to extract the texture features of COVID-19 chest CT scans. In this technique, the fractal dimensions are computed from the stack of binary images. The technique works in two steps: (1) image partitioning into stack of binary images using pair threshold binary decomposition (PTBD), (2) fractal analysis of each binary image based on boundaries, pixel count, mean gray level. 
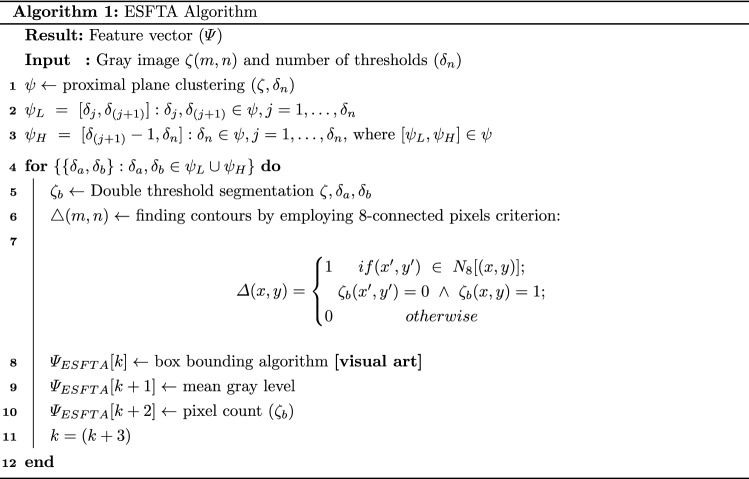


### Statistical features

Generally, data follow a normal distribution, which describes how the values of a variable are distributed. In case of normal distribution, it has two fundamental parameters; the mean and standard deviation. Observing a chest CT scan image under *M* modalities, with an assumption that all the images are spatially registered, i.e., healthy images are same and pixels correspond to the same location. From a pool of images, $x_i$, where $i = \{1,2,\dots , n\} \in X$, each of the healthy image follows a Gaussian distribution $\varPi (\lambda , \sigma )$. One observes a noticeable change in the distribution when provided an infected COVID-19 chest CT scan image. By following the underlying concept, two statistical parameters, entropy and skewness, are selected based on their vast applications in the field of machine learning.

#### Skewness

It measures asymmetry of the probability distribution about its mean. The skewness value can be negative, zero or positive. If the value is negative then the distribution curve spreads out more to the left of the mean, whereas, in case of positive value, it leans toward the right. The skewness distribution is described using the relation:

7 $$\begin{aligned} \varsigma = \frac{E(x-\mu )^3}{\sigma ^3} \end{aligned}$$

where $\mu$ and $\sigma$ are the mean and standard deviation of a random variable *x*, and *E*(*t*) denotes the expected value.

#### Entropy

Entropy offers the information regarding randomness in a signal by cogitating the system’s disorder. Due to this potential, entropy, in the current perspective, offers a useful information that can be utilized in feature representation. In this framework, Shannon entropy is utilized, which significantly improves the overall accuracy, by embedding the most relevant feature information. For both COVID-19 chest CT scans $x_1, x_2, \dots , x_N \subseteq X(m,n) \in {^{\gtrdot,\ltimes}}$ contains *N* samples. The image space has $\beta$ measure with $\beta (X) = 1$, the Shannon entropy is calculated as:

8 $$\begin{aligned} {\mathbb {E}}(X) = -\sum _{k=1}^{N}x_k \log \zeta (x_k) \end{aligned}$$

where $\zeta (x_k)$ is observing probability for a particular pixel matrix/vector of X. This whole concept allows us to identify the most superior and dominant pixels with a better variation and with least correlation.

### Feature selection framework

The genetic algorithm (GA) belongs to a class of stochastic search algorithms, which on the principle of *survival of the fittest* finds the sub optimal solution from a pool of solutions. In the GA framework, the population is developed by combining a set of chromosomes, where each chromosome constitutes a possible solution. In the proposing scenario, the extracted set of features are independently plugged into the GA block. The most discriminant chromosome/solution is later selected using the proposed entropy-based fitness optimizer.

Both texture and statistical features are used to generate two pairs of chromosomes, where each chromosome represents a feature type.

9 $$\begin{aligned} \varPhi ^k({\mathbb {X}}) =\left\{ \Psi _{WL}^1(X), \Psi _{\mathrm{ESFA}}^2(X), \varPhi _{\varsigma }^3(X), \varPhi _{{\mathbb {E}}}^4(X)\right\} ; k \in {\mathbb {Z}}, \end{aligned}$$

${\mathbb {Z}} = \{1, \dots , 4\}$ is the set of bounded integers, representing a feature chromosome. The entire population is generated from each set of chromosome *c*, continuous valued vector, having $G_j$ genes. The continuous domain offers more convergence possibilities and also minimizes the probability for a generation to be stuck in a local minima.

10 $$\begin{aligned} C_j^k = \left\{ G_1^k, G_2^k,, G_3^k,, \dots , G_m^k,\right\} \end{aligned}$$

where *k* is the chromosome index in a population, and *m* is the length of a chromosome. In what follows, we present a genetic operators including proposed crossover, mutation and selection operators for a thorough technical analysis.

### Median-replacer crossover

Selecting a pair of chromosome, $C_j^1 = \{(G_1^1, G_2^1, G_3^1, \dots , G_m^1),(G_1^2, G_2^2, G_3^2, \dots , G_m^2)\}$, where $j = \{1,2,\dots ,m\}$, for an average-replacer crossover operation. Two offsprings, ($O_l^1$ and $O_l^2$), are generated as: $O_l^1 =\lambda C_j^1(1-\lambda )C_J^2$ and $O_l^2 =\lambda C_j^2(1-\lambda )C_J^1$. The median of both offsprings is later replaced using the min/max value extracted from the parent chromosomes. A max value is assigned 1 (${\mathrm{max}}\rightarrow 1$), whereas the min value is assigned 0 (${\mathrm{min}}\rightarrow 1$). A binary random sequence is generated to select min/max value from the first selected chromosome $C_j^1$, and the same procedure applies to the second selected chromosome. Based on the generated binary rand sequence, median values of both offsprings are updated. An inversion mutation is applied on the selected number of chromosomes, whereas, for the selection, both healthy and non-healthy parents are selected for the next generation on the basis of entropy-based fitness optimizer [43].

### Entropy-based fitness optimizer

To select the next-generation offsprings, the fitness of each chromosome needs to be evaluated. In this work, we developed a novel entropy-based fitness optimizer. The whole idea revolves around the fundamental property of the feature randomness calculated by the entropy function. More the entropy value is, greater the chances of a healthy chromosome. Here, the entropy calculator identifies the maximum randomness by controlling the uncertainty. For a real-valued chromosome vector $C_j^1 = \{(G_1^1, G_2^1, G_3^1, \dots , G_m^1)\}$, the Shannon entropy is calculated using the relation:

11 $$\begin{aligned} {\mathbb {E}}_\varsigma = - \sum _{q=0}^{m-1}\left( G_q^0/ \Delta G\right) ~\log _2\beta \left( G_q^0/ \Delta G\right) \end{aligned}$$

where $G_q^1$ is the gene *q* of the first chromosome.

### Feature fusion

Features fusion is a robust strategy pursued by several researchers in the field of machine learning. The original feature space, in most of the cases, does not contain sufficient information compared to the fused feature space. Therefore, in this work, we opted feature fusion strategy to generate a resultant feature vector with enriched information. All the down-sampled features from GA block are later fused by following a cascaded design. These horizontally concatenated feature vectors is later forwarded to the classification block for final labeling using Naive Bayes classifier [44].

## Results and discussion

**Fig. 5:**
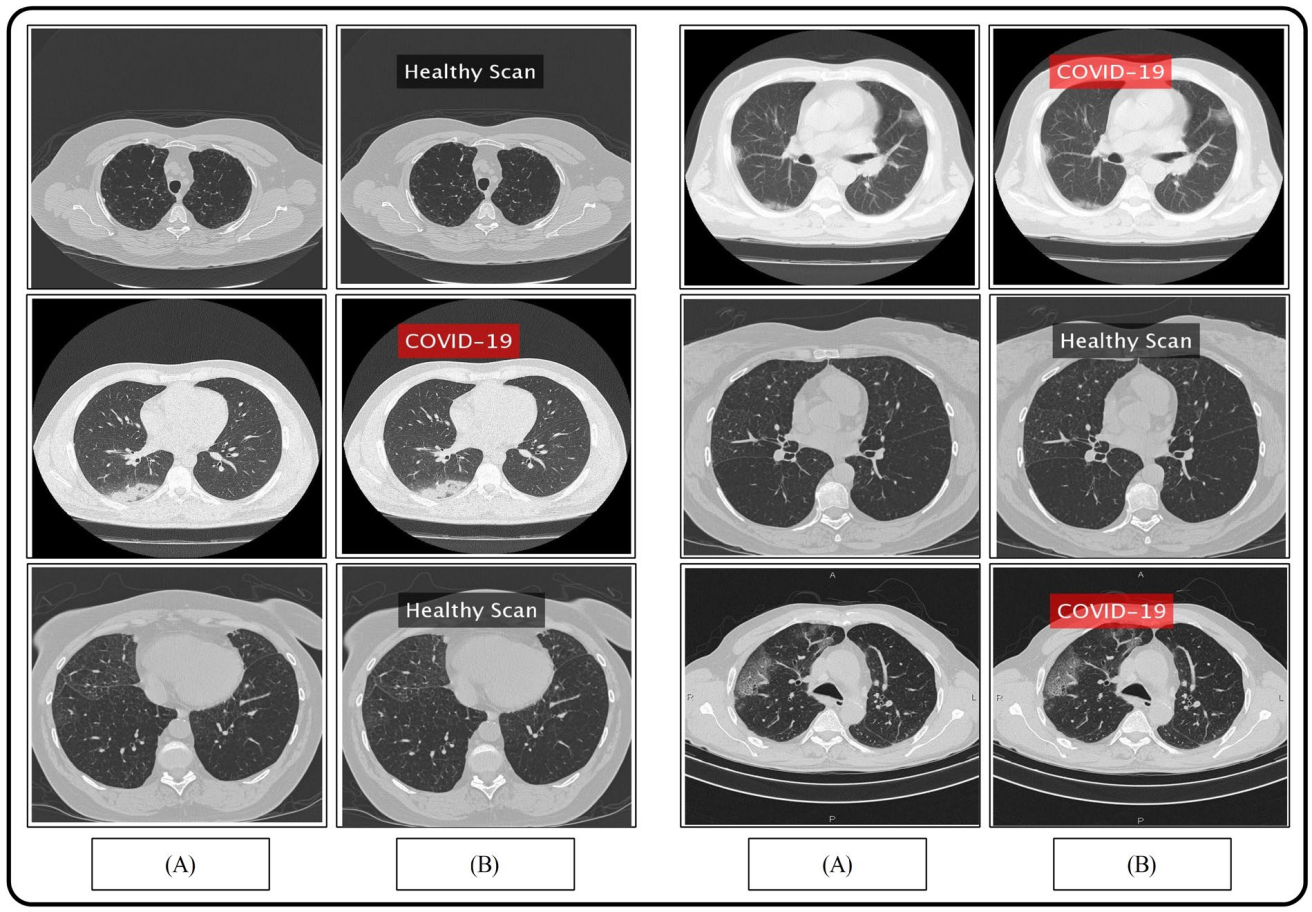
Proposed prediction results. **a** Original images; **b** proposed predicted labeled image

The proposed framework for COVID-19 pneumonia is evaluated in this section with both empirical and graphical results. For the validation, 35 subjects are considered with their Coronavirus test positive, with details provided in Sect. 3. A fair training/testing ratio of (70:30) is being followed with 70% as training data and the rest is treated as testing data. To generalize the empirical results and to recognize the precise stats, a tenfold cross-validation technique is exercised. For the final classification, a Naive Bayes classifier is selected based on its improved performance. A fair comparison is also provided with the existing state-of-the-art classifiers including fine KNN (F-KNN) [45], linear support vector machine (L-SVM) [46], ensemble bagged tree (EBT) [47] and fine tree (F-Tree) [48] classifiers. To authenticate the proposed method, several performance measures deem necessary are chosen including sensitivity (SEN), precision (PR), specificity (SPE), an area under the curve (AUC) and accuracy (ACC). The mathematical form of aforementioned measures is provided in the following equations.

12 $$\begin{aligned} {\mathrm{SEN}}&=   \frac{\delta _{\mathrm{TP}}}{\delta _{\mathrm{TP}} + \delta _{\mathrm{FN}}} \times 100\% \end{aligned}$$

13 $$\begin{aligned} {\mathrm{SPE}}&=   \frac{\delta _{\mathrm{TN}}}{\delta _{\mathrm{TN}} + \delta _{\mathrm{FP}}} \times 100\% \end{aligned}$$

14 $$\begin{aligned} {\mathrm{PR}}&=   \frac{\delta _{\mathrm{TP}}}{\delta _{\mathrm{TP}} + \delta _{\mathrm{FP}}} \times 100\% \end{aligned}$$

15 $$\begin{aligned} {\mathrm{ACC}}&=   \frac{\delta _{\mathrm{TP}}+ \delta _{\mathrm{TN}}}{\delta _{\mathrm{TP}} + \delta _{\mathrm{TN}}+\delta _{\mathrm{FP}} + \delta _{\mathrm{FN}}} \times 100\% \end{aligned}$$

where $\delta _{\mathrm{TP}}$ represents true positive, $\delta _{\mathrm{TN}}$ represents true negative, $\delta _{\mathrm{FP}}$ denotes false positive rate, and $\delta _{\mathrm{FN}}$ represents false negative rate. A few samples results are demonstrated in Fig. 5, where one can observe a binary labeling; corona positive and normal.

**Table 2:** Accuracy calculated using different feature extraction techniques

Classifier	Entropy	DWT	Skewness	SFTA	Accuracy (%)
Naive Bayes	✓				81.2
		✓			82.7
			✓		79.6
				✓	79.8
F-KNN	✓				79.8
		✓			81.6
			✓		76.5
				✓	80.2
L-SVM	✓				78.1
		✓			80.1
			✓		79.4
				✓	75.6
EBT	✓				76.3
		✓			80.9
			✓		72.6
				✓	80.4
F-Tree	✓				80.3
		✓			73.4
			✓		75.7
				✓	77.9

The results are compiled by taking into consideration three different scenarios; (1) accuracy achieved using independent features, (2) accuracy achieved after employing GA for feature selection and (3) with proposed feature selection and fusion method.

**Fig. 6:**
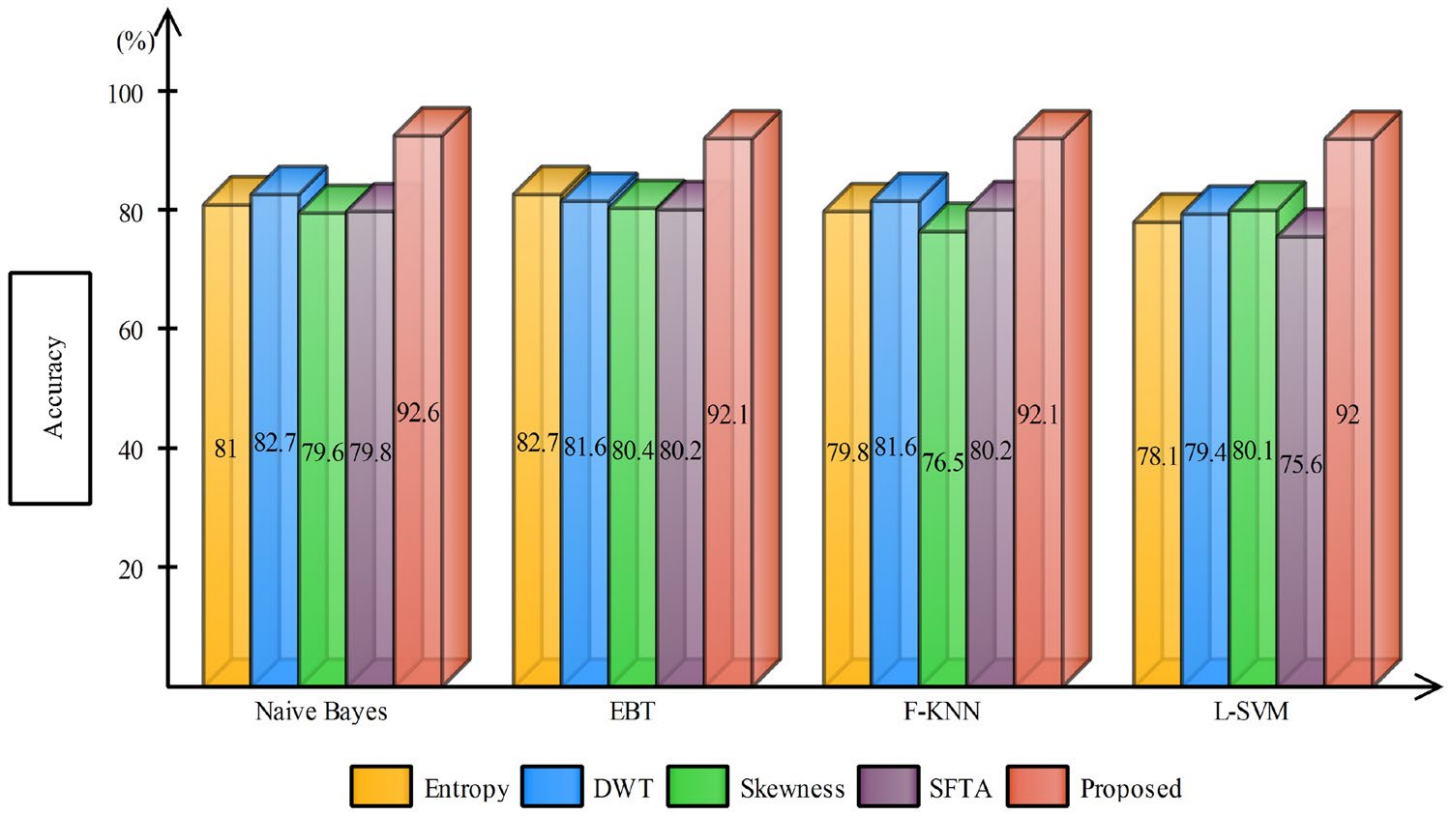
Accuracy comparison of different feature extraction techniques using bar plots

**Table 3:** Accuracy comparison of different feature extraction techniques after applying GA-based feature selection

Classifier	G-Entropy	G-DWT	G-Skewness	G-SFTA	Accuracy (%)
Naive Bayes	✓				86.7
		✓			87.4
			✓		80.4
				✓	87.7
F-KNN	✓				84.6
		✓			85.9
			✓		80.1
				✓	85.4
L-SVM	✓				84.2
		✓			85.4
			✓		78.6
				✓	82.9
EBT	✓				82.3
		✓			81.8
			✓		76.3
				✓	84.6
F-Tree	✓				81.7
		✓			83.8
			✓		79.2
				✓	78.6

Case 1 considers the features extracted independently from each technique for the classification; this is tabulated in Table 2. For a fair comparison, different classifiers are being tested and against each technique. The final results are as expected, and the Naive Bayes classifier outperforms other state-of-the-art with an average accuracy of 80.83%, while the second-best average accuracy achieved is with F-KNN (79.52%). The accuracy comparison of different feature extraction techniques with proposed is provided in Fig. 6. It is apparent from the bar plot that the independent features are of no match to the fused features for this application. One can also observe, with DWT features, most of the classifiers performed well compared to other features. This clearly shows, DWT features in this application, would be an appropriate choice compared to other features, either used solely or in the fused form.

**Fig. 7:**
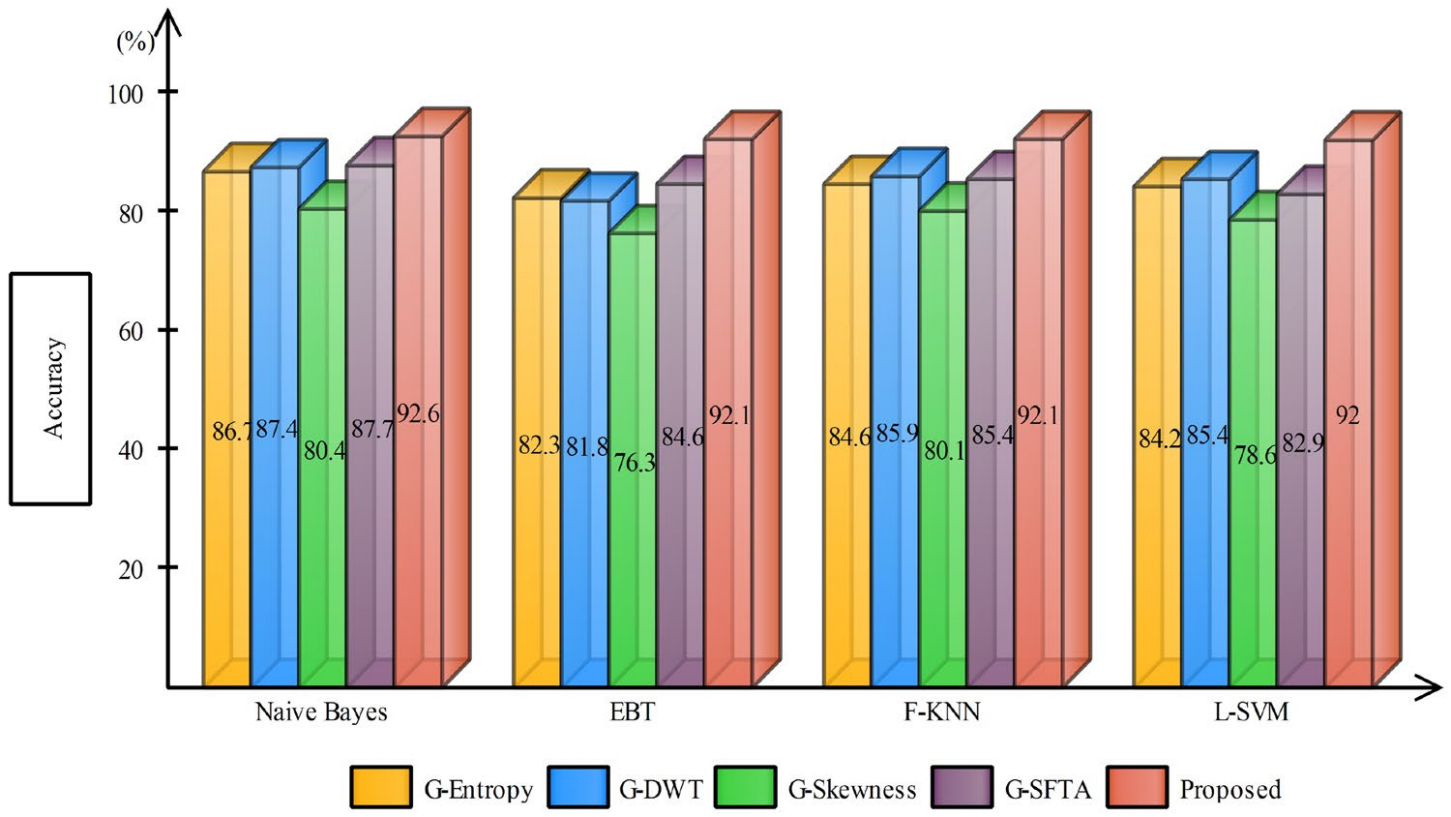
Accuracy comparison of different feature extraction techniques using bar plots after applying GA-based feature selection

In case 2, the selected features from GA are forwarded to the classifier for final labeling, Table 3. The same trend is being followed after feature selection step, and Naive Bayes classifier works exceptionally well almost for all kind features by achieving an average accuracy of $85.55\%$. F-KNN worked second best by achieving an average accuracy of $84\%$. One more time, with DWT features, classification results are exceptional with almost all the classifiers. The average classification accuracy achieved using GA-DWT features by all the selected classifiers are 84.86% compared to 83.85% using G-SFTA. Figure 7 demonstrates that the average accuracy achieved after GA increased compared to stand alone features. A vertical bar clearly indicates that the accuracy margin between the proposed and after GA selection is still comparable, which strengthens the positive significance of feature fusion.

**Table 4:** A comparison of state-of-the-art classifiers using proposed GA controlled feature selection and fusion method

Classifier	Sensitivity (%)	Precision (%)	Specificity (%)	AUC	Accuracy (%)
Naive Bayes	**92.5**	**92.5**	**92.0**	**0.99**	**92.6**
F-KNN	92.0	92.0	91.0	0.96	92.0
L-SVM	91.7	92.0	91.0	0.96	92.1
EBT	92.2	92.3	94.0	0.98	92.2
F-Tree	91.5	91.5	93.0	0.98	91.6

Significant values are shwon in bold

Using the proposed framework, the achieved accuracy using the Naive Bayes classifier is $92.6\%$, whereas a few other classifiers (EBT, L-SVM and F-KNN) behave significantly better to achieving an average accuracy of $92.2\%, 92.1\%$ and $92.0\%$, respectively. The authenticity of proposed framework is further validated from the selected performance parameters including sensitivity ($92.5\%$), specificity ($92.0\%$), precision ($92.5\%$) and the AUC (0.99), see Table 4. From the sensitivity and specificity values, it is quite obvious that the proposed framework has successfully managed to achieve a high true positive and negative rates by correctly classifying the actual positive and actual negative samples. To further describe the performance of a classifier on a set of test data, a confusion matrix is provided, Table 5. From the stats, one can develop a clear understanding, that out of total test samples, $93\%$ are correctly labeled as COVID-19 infected, whereas around $7\%$ are misclassified as normal.

**Table 5:** Confusion matrix of Naive Bayes classifier after applying optimized GA for features selection

COVID-19	93%	7%
Normal	8%	92%
	COVID-19	Normal

**Fig. 8:**
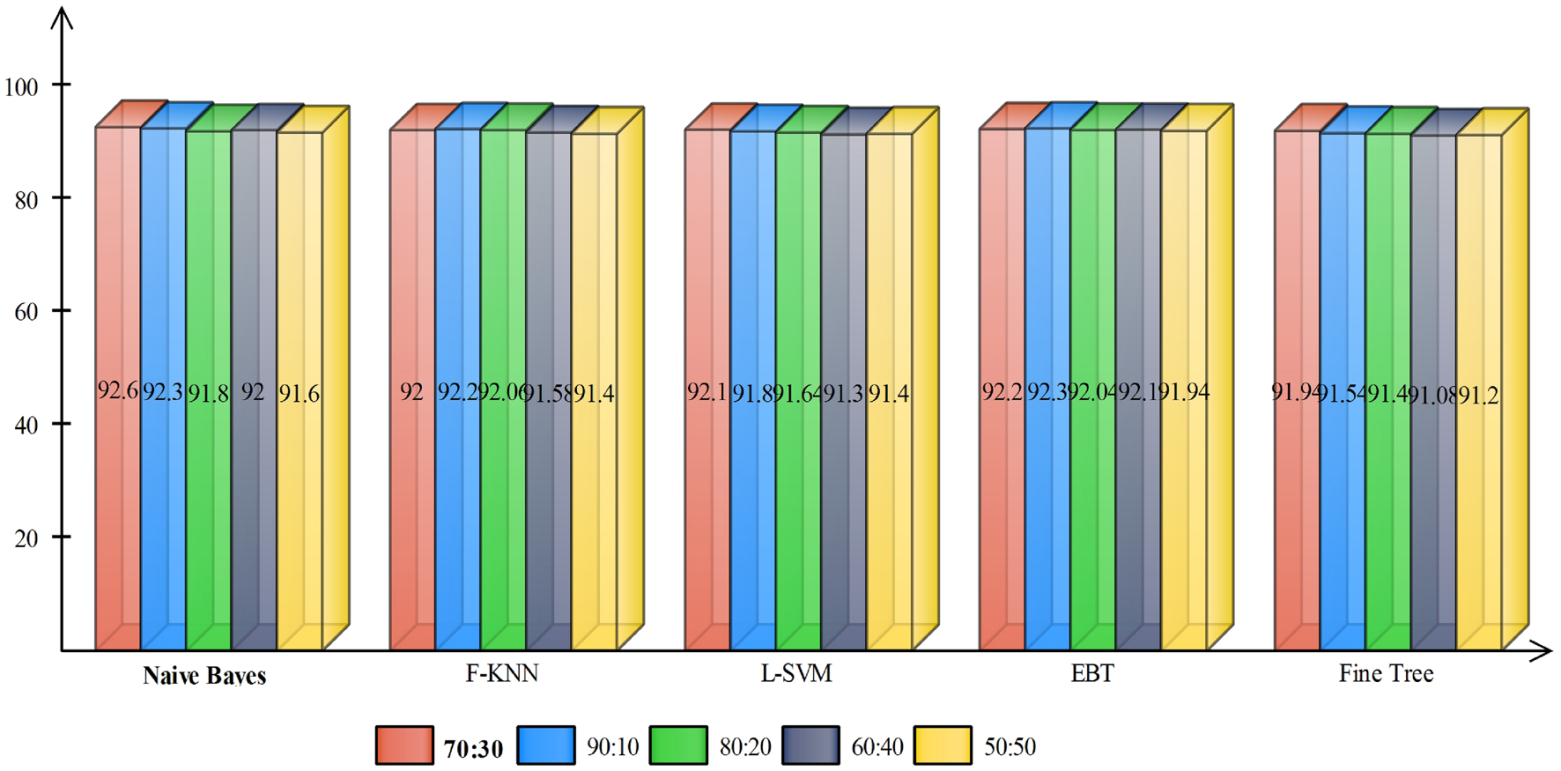
Comparison of proposed results on different training and testing sets

In addition, we compare the proposed method results on different training and testing samples such as 70:30, 60:40, 90:10 and so on. The results are plotted in Fig. 5. In this figure, it is shown that the 90:30 approach results are fine but if we consider the standard process of validation, then 70:30 approach results are more useful (Fig. 8).

## Statistical significance

The objective here in performing the statistical analysis is to gain a high level of confidence in the proposed method. The results are statistically significant, if they are likely not caused by chance. We employed the analysis of variance (ANOVA) to demonstrate, that either the results are statistically significant or not. In this work, we consider the proposed scenario for three different classifiers (Naive Bayes, EBT, L-SVM)—selected on the basis of their improved performance compared to the rest. A Shapiro–Wilk test is performed for assumption of normality, while Bartlett’s test—for homogeneity of variance with a significance level $\alpha = 0.01$. The means of our approach are $\bar{x}_1$, $\bar{x}_2$, and $\bar{x}_3$—calculated from the overall accuracy of both classifiers. The null hypothesis $H_0$, given that $\bar{x}_1 = \bar{x}_2 = \bar{x}_3$, while the alternative hypothesis $H_a$ given that $\bar{x}_1 \ne \bar{x}_2 \ne \bar{x}_3$. We computed the *p* value and tested the null hypothesis, $H_0$, if it is rejected, $p < \alpha$, then we will be applying Bonferroni post hoc test.

**Fig. 9:**
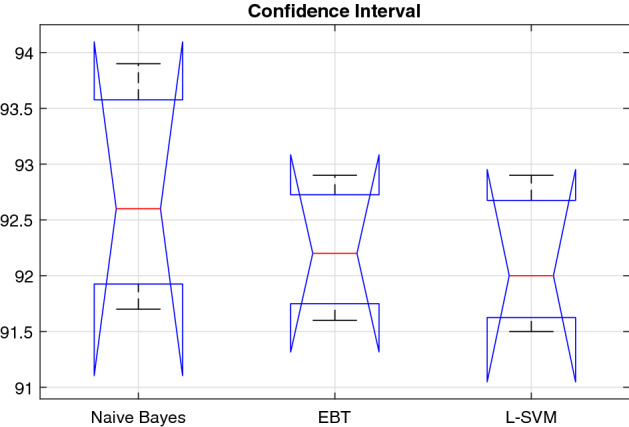
Box-plot of accuracy values on the selected classifiers (1: SVM-C, and 2: SVM-Q)

For the proposed entropy controlled GA method (E-GA), and with selected classifiers (Naive Bayes, EBT and L-SVM), the Shapiro–Wilk test generated *p* value, $p_u = 0.8002$, $p_v = 0.9152$, and $p_l = 0.6878$. By following the Bartlett’s test, the associated Chi-squared probabilities are: $p_u = 0.371$, $p_v = 0.339$, and $p_l = 0.410$. From the calculated *p* values of two different classifiers, which are significantly greater than $\alpha$. Therefore, from the test (normality and equality of variances), we failed to reject the null hypothesis $H_0$, and confirm that the data were distributed normally, and their variances are homogeneous. ANOVA test including five different parameters (degree of freedom (*df*), a sum of squared deviation (SS), mean squared error (MSE), *F*-statistics, and *p* value) is shown in Table 6. The performance range of three selected classifiers based on the proposed method is shown in Fig. 9.

**Table 6:** ANOVA test on two selected classifiers based on the proposed method

Variance source	SS	*df*	MSE	*F*-Statistics	*p* value
Between	0.6212	1	0.313	0.431	0.667
Within	4.324	4	0.716	–	–
Total	4.924	5	–	–	–

The results are also validated by utilizing Bonferroni post hoc test, which is the most common approach to be applied whenever there exists a chance of a significant difference between the means of multiple distributions. It was certified that the proposed method performed better compared to several existing methods.

## Conclusion

A computerized technique is proposed in this work for the prediction of COVID-19 from the CT scans. Textural and statistical features are extracted from raw CT images, and then, only best features are selected based on optimized genetic algorithm. The selected features are serially concatenated and later classified using the Naive Bayes classifier. The experimental process is performed on the collected COVID-19 positive and healthy samples and shows the proposed method to be effective. The main contribution of this work is an optimized genetic algorithm for best selection. Using this algorithm, the accuracy of individual feature type is improved and when all selected features are combined, then a significant change has been observed in the accuracy. Based on the performance of this algorithm, we concluded that the selection of most relevant features improves the accuracy, but on the other side, it is a high chance that we miss the important features that play a contribution in the improvement of prediction accuracy. Also, this problem may occur when we have more patients data for final testing. Therefore, in the future studies, we will focus on the reduction of these features.
